# Genetic, morphological and ecological variation across a sharp hybrid zone between two alpine butterfly species

**DOI:** 10.1111/eva.12925

**Published:** 2020-02-07

**Authors:** Thibaut Capblancq, Laurence Després, Jesús Mavárez

**Affiliations:** ^1^ Laboratoire d’Écologie Alpine UMR UGA‐USMB‐CNRS 5553 Université Grenoble Alpes Grenoble France; ^2^ Department of Plant Biology University of Vermont Burlington VT USA; ^3^ Departamento de Ciencias Biológicas y Ambientales Universidad Jorge Tadeo Lozano Bogotá Colombia

**Keywords:** Alps, *Coenonympha*, genetic cline, hybrid zone, morphometrics, speciation

## Abstract

Identifying the mechanisms involved in the formation and maintenance of species is a central question in evolutionary biology, and distinguishing the selective drivers of populations’ divergence from demographic processes is of particular interest to better understand the speciation process. Hybrid zones are recognized to provide ideal places to investigate the genetic architecture of speciation and to identify the mechanisms allowing diverging species to maintain their integrity in the face of gene flow. Here, we studied two alpine butterfly species, *Coenonympha macromma* and *C. gardetta,* which can be found flying together and hybridizing in narrow contact zones in the southern French Alps. We characterized the genomic composition of individuals, their morphology and their local habitat requirements, within and around a hybrid zone. Genetic diversity analysis at 794 SNPs revealed that all individuals within the hybrid zone were highly admixed, which was not the case outside the hybrid zone. Cline analysis showed that, despite ongoing hybridization, 56 out of 122 loci differentially fixed or nearly so between the two species were impermeable to introgression across the sharp hybrid zone (9 km wide). We also found concordance in cline position and width among genetic, morphological and environmental variation, suggesting a coupling of different reproductive barriers. Habitat characteristics such as the presence of trees and shrubs and the start of the growing season were strongly associated with the genetic variation, and we found evidence of divergence at genetic markers associated with morphology and physiology, putatively involved in visual or environmental reproductive isolation. We discuss the various behavioural and ecological factors that might interplay to maintain current levels of divergence and gene flow between this species pair.

## 1 INTRODUCTION

Hybrid zones are geographic regions where genetically divergent taxa meet and hybridize (Barton & Hewitt, 1985). They are maintained by a balance between divergent selection, promoting species differentiation, and gene flow, acting as a homogenizing force between genetic pools (Barton, 2001; Barton & Hewitt, 1985; Endler, 1977; Haldane, 1948; Jiggins & Mallet, 2000). In such zones, genes can potentially be exchanged between populations through segregation and recombination in hybrids, at rates that, depending on the intensity of selection, can go from virtually zero for the genomic regions highly involved in species isolation and differentiation, to almost free circulation for the “neutral” or not divergently selected parts of the genome (Barton, 1979; Harrison, 1986, 1993; Payseur, 2010; Rieseberg, Whitton, & Gardner, 1999). Hybrid zones thus provide natural segregation experiments that allow both the study of factors that shape gene flow between divergent lineages and the identification of genetic loci and traits that contribute to species isolation (Payseur, 2010). The analysis of hybrid zones has therefore played an important role in the understanding of the genomic regions under selection and/or responsible for isolation in species that are difficult to breed in the laboratory (e.g. insects with a diapause) or long‐lived organisms (e.g. trees) (Derryberry, Derryberry, Maley, & Brumfield, 2014; Lindtke et al., 2012).

The estimation of cline width and slope for a genetic locus along the hybrid zone provides important information about its introgression rate. When performed over many loci distributed throughout the genome, this analysis not only gives genome‐wide introgression rates, but also allows for the identification of genomic regions exhibiting significantly higher‐than‐average or lower‐than‐average introgression between species (Abbott et al., 2013; De La Torre, Ingvarsson, & Aitken, 2015; Gay, Crochet, Bell, & Lenormand, 2008). The study of such patterns of heterogeneity in genetic exchange along the genome can help identify barrier loci, and when analysed together with morphological and behavioural traits, or with environmental variables, may shed light on the forces underlying species isolation and differentiation (Lexer, Buerkle, Joseph, Heinze, & Fay, 2007; Lindtke et al., 2012). Nonetheless, untangling the drivers of species differentiation and their genomic consequences can be difficult. The main reason is that hybrid zones tend to be positioned in areas of low population density and/or environmental ecotones (Endler, 1977; Leaché, Grummer, Harris, & Breckheimer, 2017; Taylor et al., 2014; Wielstra et al., 2017), even when environmental factors are not the main drivers of species isolation (Bierne, Welch, Loire, Bonhomme, & David, 2011). Besides, a hybrid zone associated with intrinsic reproductive isolation factors (e.g. genetic incompatibilities, assortative mating) may move and stabilize around an extrinsic natural barrier or ecotone, which may erroneously suggest a role for environmentally driven selection in the formation of the hybrid zone (Barton & Hewitt, 1985; Hewitt, 1988). In fact, the coupling of intrinsic and extrinsic isolating factors interacting along hybrid zones may be relatively common in the wild (Abbott et al., 2013; Bierne et al., 2011). Coincidence of barrier effects can occur either via selection on individual barrier effects or as a by‐product of demographic processes. It can further evolve as an adaptive response to indirect selection, through the evolution of prezygotic barriers to gene flow in response to costs of hybridization (reinforcement) (Butlin & Smadja, 2017).

Here, we investigate the strength and dynamics of isolation in a contact zone between two alpine butterfly species in the genus *Coenonympha* (Nymphalidae, Satyrinae): the Alpine Heath *C. gardetta* (Prunner 1798) and the Darwin Heath *C. macromma* (Turati & Verity 1910). *Coenonympha gardetta* is a typical alpine species, largely distributed at elevations above 1,500 m along the Alps, the French Massif Central and the Balkans, whereas *C. macromma* is restricted to the south part of the French Alps, where it replaces *C. gardetta*. The two species are found flying together in narrow zones where their distribution ranges abut. Hybrids are relatively common in these contact zones, which highlights incomplete reproductive isolation between species, even if they remain highly differentiated genetically at a large geographic scale (Capblancq, Mavárez, Rioux, & Després, 2019). Identifying the mechanisms involved in reproductive isolation between these species would provide a better understanding of the speciation process for *C. macromma,* which has been suggested to be the product of homoploid hybridization between *C. gardetta* and a third species, the Pearly Heath *C. arcania* (Linné 1761). Indeed, in a previous study (Capblancq, Després, Rioux, & Mavárez, 2015), we showed that *C. macromma* is composed of a genetic mixture between *C. gardetta* and *C. arcania* (30% and 70%, respectively) and that it exhibits intermediate wing morphology and ecological preferences (climatic niche). Statistical modelling of different past evolutionary scenarios allowed us to confirm the hybrid origin of *C. macromma* and estimate its emergence at around 12,000–20,000 years ago. Investigating the patterns of species divergence and its drivers in this clade would further provide more general insights into the process of homoploid hybrid speciation (Mavárez & Linares, 2008; Schumer, Rosenthal, & Andolfatto, 2014).

We used genomic, morphological and environmental data to investigate the fine‐scale divergence between *C. gardetta* and *C. macromma*. After identifying an active and narrow hybrid zone between these two species, we performed a geographic cline analysis to characterize the genetic architecture of the hybrid zone and to investigate the genomic heterogeneity of introgression between the two species. We also looked for patterns of morphological and ecological divergence between species. Our goal was to evaluate the degree of concordance between ecological, morphological and genetic clines in the hybrid zone and to identify loci and traits potentially involved in species divergence and isolation.

## 2 MATERIALS AND METHODS

### 2.1 Sample collection

**Figure 1:**
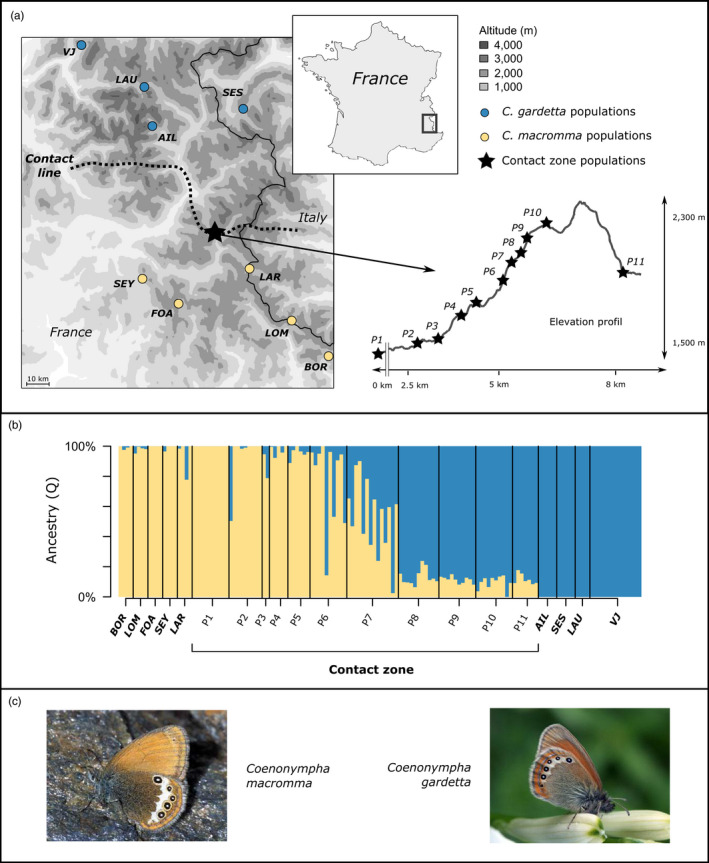
(a) Map showing the contact line between *Coenonympha macromma* and *C. gardetta* ranges in the French Alps, the location of sampled populations and the profile of the contact zone. (b) sNMF plot showing ancestry scores for all the individuals at *K* = 2, indicating genetic contributions from *C. macromma* or *C. gardetta* genetic clusters across the sampling zone. (c) Pictures of the two parental species, *C. gardetta* (Photo by Claire Hoddé, https://www.lepinet.fr) and *C. macromma* (Photo by Tristan Lafranchis, https://www.lepinet.fr)

We sampled 142 butterflies from 20 sites in the southern French Alps: 11 sites from a *C. gardetta*/*C. macromma* contact zone in which interspecific hybrids seem to be common and 9 sites located outside this area within the same region (Figure 1 and Table S1). The contact zone sites (P1 to P11; Figure 1) are distributed along an 8‐km transect (P1 at position 0 m, P11 at position 8,100 m) located near the Col de Vars, a high‐elevation alpine pass (2,100 m) situated where the ranges of *C. gardetta* (north) and *C. macromma* (south) overlap. Elevation and habitat change markedly along this transect, from mid‐elevation semi‐closed vegetation habitats (i.e. a high coverage by shrubs and trees) at P1, to high‐elevation open vegetation (grasslands) at P11. The 9 sites sampled outside the contact zone have individuals with wing traits characteristic of only one of either parental species (*C. gardetta*: LAU, SES, AIL, VJ; *C. macromma*: SEY, LAR, LOM, FOA, BOR; Figure 1 and Table S1). Sampled individuals were kept in glassine envelopes in the field and in ethanol 96% at −20°C in the laboratory until DNA extraction.

### 2.2 Genetic data acquisition

DNA was extracted from the complete thorax of each individual using the DNeasy Blood and Tissue Kit (Qiagen, Germany). A data set of single nucleotide polymorphisms (SNPs) was produced using a double‐digested restriction site‐associated DNA (ddRAD) sequencing procedure using a modified version of the protocol of Peterson, Weber, Kay, Fisher, and Hoekstra (2012) as described in Capblancq et al. (2015). Five different *Sbf*I/*Msp*I ddRAD libraries were sequenced, each one in 1/10 of lane of a HiSeq 2,500 Illumina sequencer (Fasteris S.A., Switzerland). The obtained DNA reads (~100 million of 2 × 125 paired‐end reads) were used to call SNP genotypes with the *STACKS* pipeline (Catchen, Hohenlohe, Bassham, Amores, & Cresko, 2013) using a Phred score of 10 for read filtering (*process_radtags* function), a minimum coverage of 4 to create a stack (‐*m* 4 in *ustacks* function) and a maximum of 6 different nucleotides to merge two different stacks (‐*M* 6). Highly repetitive stacks and overmerged tags were dropped using both the “Removal algorithm” and the “Deleveraging algorithm.” Furthermore, a maximum of 8 mismatches was allowed for considering two individual tags as the same locus and to merge them in the catalogue (‐*n* 8 in the *cstacks* function). In order to evaluate the experimental success for each library and each individual, we used *ProcessMyRAD* scripts (https://github.com/cumtr/PmR, Cumer et al., 2018) to produce graphical outputs at different steps of the treatment. This pipeline provides the proportion and number of reads retained after quality filtering, sequencing nucleotide quality along the reads, and the proportion of the different nucleotides. It also allows repeating the critical step of de novo individual locus assembly (*ustacks*) using a range of M values. By looking at the number of assembled RAD tags across *M* values, it provides a way to find a value allowing assembling a maximum of loci without stacking too many paralogs. *M* = 6 was chosen because values above this threshold did not substantially increase the number of assembled loci within individuals (Figure S1).

To avoid an excess of missing data, all further analyses were performed using only RAD fragments genotyped for more than 40% of the samples of each of the three types of population (pure *C. gardetta*, pure *C. macromma* and contact zone) (parameters ‐*r* 0.4 and ‐*p* 3 in the *populations* function of STACKS). Only one polymorphic site was randomly kept for each polymorphic RAD fragment to avoid inclusion of physically linked SNPs. Finally, SNPs with a minor allele frequency lower than 1% (<2 individuals) were removed from the data set.

### 2.3 Genetic diversity and structure across the populations

Genetic diversity statistics, *that is* observed heterozygosity (*H*_obs_), gene diversity (*H*_exp_) and *Fis*, were calculated for populations with at least 5 samples, using the function “*basic.stats”* of the *hierfstat* R package (Goudet & Jombart, ). Population structure across the whole study area was assessed using sNMF, a constrained method of genetic clustering (see Frichot, Mathieu, Trouillon, Bouchard, & François, 2014). This analysis was performed using the R package *LEA* (Frichot & François, 2015), imposing two genetic clusters (*K* = 2) in order to estimate the probability of assignation of each individual to the two parental species genetic groups (*C. macromma* and *C. gardetta*). We assumed that the pure parental genetic makeup was correctly inferred in the analysis by using numerous (20 *C. macromma* and 39 *C. gardetta*) individuals from species‐specific allopatric populations (Figure 1).

### 2.4 Individual index of hybridization and interspecific heterozygosity estimation across the contact zone

Genetic admixture was estimated by maximum likelihood for each individual within the contact zone using the procedure implemented in the R package *introgress* (Gompert & Alex Buerkle, 2010). This procedure provides a genetic hybrid index (HINDEX) representing an estimate of the proportion of alleles inherited from one of the two parental species (Buerkle, 2005). The HINDEX value thus estimates a global index of admixture taking all the loci into account. Reference values for allele frequencies were established for each parental species from pure populations of *C. macromma* or *C. gardetta* (Figure 1 and Table S1). The contact zone was analysed considering all individuals as potential hybrids between the two parental allele frequency references. The HINDEX ranges from 0 to 1, with extreme values corresponding to pure individuals of each reference parental species. This method allows for the use of codominant markers and, quite appropriately for closely related species, for markers that are not necessarily fixed between taxa (Buerkle, 2005).

Interspecific heterozygosity was also estimated for each individual within the contact zone by using the function *calc.intersp.het* of the *introgress* R package, which calculates the proportion of an individual's genome with alleles inherited from each parental species (Buerkle, 2005). It gives an estimation of the direct bi‐ancestrality of each sampled genotype. This method specifically estimates the heterozygosity due to admixture between the two defined parental groups, meaning that an F1 individual would return a value close to 1 (at least > 0.85 according to Milne & Abbott, 2008). Interspecific heterozygosity is thus designed to detect recent hybrids (e.g. F1, F2, backcrosses), as they are expected to show index values higher than parental individuals and ancient hybrids. Indeed, in populations where admixture took place a long time ago and with no contemporary interspecific gene flow, individuals are expected to show levels of interspecific heterozygosity similar to parental populations, but with a mosaic of alleles from either reference population (De La Torre et al., 2015; Fitzpatrick, 2012; Lindtke et al., 2012).

### 2.5 Genetic clines along the contact zone

To further investigate the genetic transition between *C. macromma* and *C. gardetta* along the contact zone, we fitted a genetic cline model to the loci with differences in allele frequencies between parental populations >0.8, using the *hzar* R package (Derryberry et al., 2014). We only analysed those highly divergent loci because cline analysis aims at looking at the change along the contact zone of features (genes or traits) characteristic of each parental population. We found that 122 SNP (hereafter “diagnostic loci”) fulfilled the minimum difference condition (see Results and Figure S2). The clines for the diagnostic loci were fitted with the 15 models designed by Derryberry et al. (2014). These 15 models are combinations of three possible allele frequency intervals (*p*_min_–* p*_max_ set to the observed values, fixed to 0 and 1, or estimated during the procedure) and five possible ways to fit tails (none fitted, left only, right only, mirror tails and both tails estimated separately). To ensure that the likelihood surface was correctly explored, we conducted three independent runs for each model, each run consisting of 20,000 iterations following an initial burn‐in phase of 500 iterations. For each locus, we retained the model with the lowest Akaike information criterium (AIC) and extracted from this model the parameters for cline width (*w*) and centre (*c*) estimates. We also used the difference between the observed minimum and maximum allelic frequencies (∆*P*) to estimate the slope (*m*) of each cline using the following equation: *m* = (∆*P*/*w*) (Stankowski, Sobel, & Streisfeld, 2017).

### 2.6 Morphological variations between parental species and along the contact zone

To identify whether the genetic transition between species, especially across the contact zone, was correlated with morphological variation, we performed geometric morphometric analyses of wing shape, patterns and venation. We focused on traits that are already known to discriminate between *C. macromma* and *C. gardetta*, such as wing size, the position of eyespots and the shape of the hindwing white band (see Capblancq et al., 2015). We used 22 and 18 homologous landmarks in the fore‐ and hind‐wings, respectively, corresponding to vein intersections and termini, eyespot centres and the outline of the hindwing white band (Figure S3). The 80 coordinates resulting from these 40 landmarks were used to perform Procrustes superimposition in order to remove nonshape variation in location, scale and orientation. Our goal here was to identify different morphological traits that discriminated the two parental populations of *C. macromma* and *C. gardetta* and then analyse the variation of these traits across the contact zone. We thus conducted a series of Procrustes superimposition on different sets of landmarks corresponding to four different traits: the vein intersections and termini of the forewing as a proxy of forewing global shape, the vein intersections and termini of the hindwing as a proxy of hindwing global shape, the eyespot landmarks as a proxy for eyespot positions, and the white band landmarks as a proxy for white band shape. We then performed for each trait a principal components analysis (PCA) on the superimposed coordinates and used the two first PCs to visualize and test for the potential divergence between parental populations of *C. macromma* and *C. gardetta*. We kept only the two first PCs for each trait because they gathered in each case a high proportion of the morphological variation (see Results), and we wanted to stay focused on the strongest interspecific morphological divergences between parental species. The trait differences between parental populations were tested using a one‐way MANOVA on the first two PCs, with shape as the dependent variable and species as a factor. A wing size index was also obtained using the sum of the log‐transformed centroid sizes estimated independently for the fore‐ and hindwing landmarks. Difference in size among species was tested with Student's *t* test with size as dependent variable and species as factor. The generalized Procrustes analysis and the different statistics were performed in R with the packages *Rmorph* (Baylac, 2012), *ade4* (Chessel, Dufour, & Thioulouse, 2004) and *APE* (Paradis, Claude, & Strimmer, 2014).

We then fitted seven cline models to the three morphological traits distinguishing parental species in the geometric morphometric analysis: wing size, the position of hindwing eyespots and the shape of the forewing white band (Figures S3 and S4). We fitted a cline model to these three traits with the *hzar* package and using the sum of the log‐transformed centroid sizes for the fore‐ and hindwing as a proxy for the global wing size, the loadings of each individual along the first principal component (PC1) remaining from the Procrustes superimposition of the eyespot landmarks as a proxy for eyespot positions, and the loadings of each individual along the first principal component (PC1) remaining from the Procrustes superimposition of the white band landmarks as a proxy for white band shape. PC1 represented most of the variation for the two last traits (66 and 47%, respectively) and discriminated the parental populations (see Results). The seven models used were derived from the ones described for morphological traits in Derryberry et al. (2014) and are characterized by: a trait interval fixed to the observed values with five possible combinations of fitting tail (none fitted, left only, right only, mirror tails and both tails estimated separately) or a trait interval estimated during the procedure with two possible combinations of fitting tail (none fitted or both tails estimated separately). We then selected the best‐fit model based on AIC and recovered from it the centre, width and slope of the cline.

The association between morphological traits and the individual genetic composition was tested through linear regression with HINDEX as response variable and either wing size, PC1 scores of eyespot alignment or PC1 scores of white band shape as explanatory variable. All regressions were performed in R. The strength of the association represented by the r‐squared of the linear regression was compared among traits.

### 2.7 Characterization of ecological clines

To identify whether the genetic transition between species, especially across the contact zone, was correlated with environmental variation, we analysed ecological differences among populations using four climatic variables and three habitat variables. The four climatic variables were recovered from the WorldClim database (Hijmans, Cameron, Parra, Jones, & Jarvis, 2005, http://worldclim.org): annual mean temperature, annual precipitation, precipitation seasonality and temperature seasonality. Two habitat descriptors were obtained from the analysis of temporal series of NDVI (Normalized Difference Vegetation Index) rasters from MODIS satellite imagery every 16 days from 2000 to 2015 (Choler, 2015): the start date of the growing season and the maximum NDVI of the year, which is a proxy for primary productivity (Choler, 2015). The proportion of high vegetation structures (trees and shrubs) was extracted, for parental populations, from Corine Land Cover rasters of habitats (http://land.copernicus.eu/pan-european/corine-land-cover/clc-2012) in a pixel of 250 m × 250 m around each sampled population. For the eleven localities within the contact zone, we re‐estimated the proportion of high vegetation structures by sampling in each site three plots of 10 m^2^ where at least one *Coenonympha* individual was found. On these plots, we counted the number of times a tree or a shrub is present at each of the 60 points distributed each 30 cm along the two diagonals. Difference in environmental conditions between species was tested with Student's *t* test with each of the environmental variables as dependent variable and species as factor, using the environmental values of each pure species locations: 5 sites for *C. macromma* and 4 sites for *C. gardetta* (Figure 1).

We fitted the same seven cline models as for morphological traits to the environmental variables showing a significant divergence between pure species populations (see above). Again, we selected the best‐fit model based on AIC and recovered from it the cline centre, width and slope. The association between environmental variation and genetic composition of individuals was tested through independent linear regression with HINDEX as response variable and each environmental variable as explanatory variable. All the regressions were performed in R, and we recovered the strength of the association represented by the r‐squared of the linear correlation to compare them among variables.

## 3 RESULTS

### 3.1 Genetic data

The ddRAD libraries produced ~ 100 million reads, 80% of which were used for further analyses owing to their high read quality (>30) and uniform distribution of nucleotides along the read (Figure S5). These data allowed the assembly of 3,200 RAD fragments per individual on average, with a mean coverage of 35 reads/fragment for the 142 individuals analysed. The influence of the clustering parameter M on fragment reconstruction was visually inspected and guided the choice of *M* = 6 (Figure S1). A total of 899 RAD fragments were polymorphic, representing a total of 5,123 SNPs. However, retention of polymorphic RAD fragments that were present in at least 40% of the individuals in each of the three populations, and random selection of 1 SNP per fragment (maf > 0.01), resulted in a final data set of 794 SNPs.

### 3.2 Genetic characterization of the contact zone

**Figure 2:**
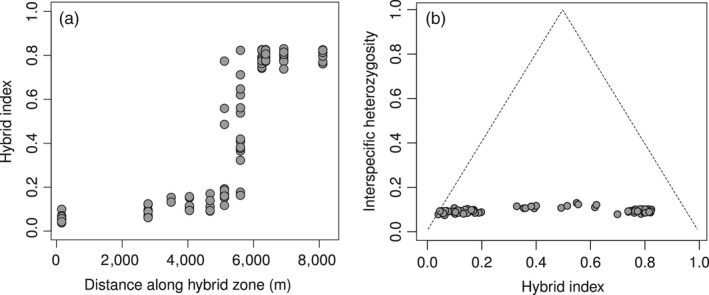
(a) Individual HINDEX estimates across the eleven populations constituting the contact zone between *Coenonympha macromma* and *C. gardetta* in the French Alps. (b) Interspecific heterozygosity (the fraction of markers heterozygous for alleles from both species) versus HINDEX

The samples from the *C. macromma/C. gardetta* contact zone were highly admixed*,* whereas the allopatric populations of each species showed genetically pure individuals (Figure 1). The highest proportion of admixture was found in populations P6 and P7, in which almost only admixed individuals with variable ancestry proportions were collected. We observed a rapid decrease in the genetic contribution of *C. gardetta* from populations P1 to P5, with only genetically pure *C. macromma* individuals in P1 (Figure 1b). At the opposite, although the *C. macromma* genetic contribution strongly decreased from populations P8 to P11, a genetically pure *C. gardetta* was not observed, as individuals in P10 and P11 still retained a *C. macromma* genetic contribution of about 15%. The HINDEX analysis showed a similar pattern with estimates ranging from almost 0.06 in P1 to 0.84 in P11 and with intermediate values for P6 (0.3) and P7 (0.5) (Table 1). Furthermore, the variance of individual HINDEX estimates increased greatly within the two populations at the core of the hybrid zone (P6 and P7) in comparison with the other populations (Figure 2a).

**Table 1:** Table of sample size, genetic diversity estimates and mean HINDEX for the eleven populations sampled in the contact zone between *Coenonympha macromma* and *C. gardetta*

Population	*N*	*H*_obs_	*H*_exp_	*F*_IS_	*H*_index_
*C. macromma*	20	0.082	0.162	0.422	0
P1	10	0.082	0.153	0.352	0.06
P2	9	0.098	0.173	0.329	0.08
P3	2	–	–	–	0.14
P4	5	0.092	0.164	0.306	0.13
P5	6	0.102	0.171	0.306	0.12
P6	10	0.104	0.200	0.387	0.30
P7	14	0.118	0.197	0.340	0.50
P8	11	0.101	0.159	0.290	0.83
P9	10	0.102	0.160	0.294	0.81
P10	10	0.107	0.171	0.301	0.82
P11	7	0.098	0.161	0.298	0.84
*C. gardetta*	39	0.086	0.133	0.344	1

In parallel, we observed only a slight increase in genetic diversity (*H*_exp_) and heterozygosity (*H*_obs_) within the hybrid zone compared to pure populations of parental species (Table 1). The unexpected small increase in heterozygosity, considering the mixing of two divergent genetic backgrounds in this area, was confirmed by the *introgress* analysis, which returned a very low interspecific heterozygosity for the individuals sampled in the contact zone, even for the ones exhibiting the highest values of admixture (Figure 2b). The low levels of interspecific heterozygosity indicate that only a low proportion of loci per individual were inherited from the two parental populations. This means that the intermediate HINDEX values observed for many individuals in the contact zone were more likely due to a mosaic of parental contributions in the genome than to recent hybridization.

### 3.3 Genetic cline analysis

**Figure 3:**
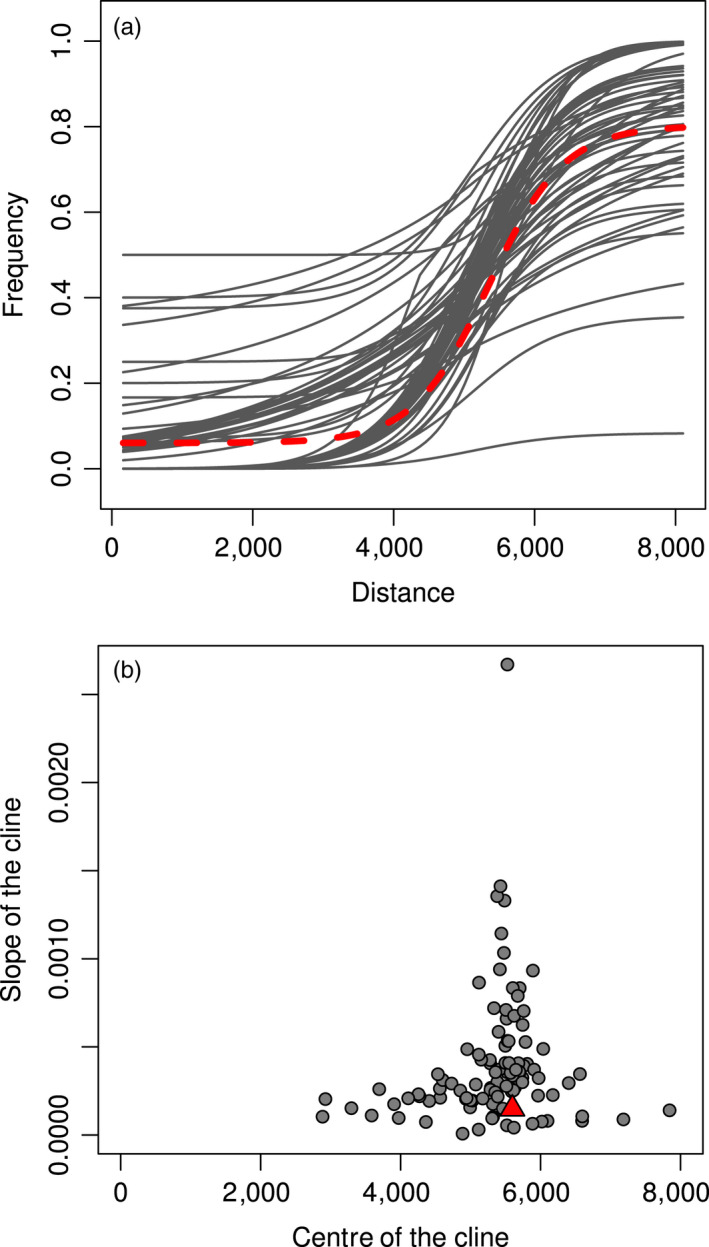
Plot of the maximum‐likelihood cline for the best‐fit model from the fifteen‐model comparison for all the 122 species‐specific loci (a). The red trait represents the HINDEX maximum‐likelihood cline. (b) Relationship between cline centre and cline slope estimates from the ML cline of each locus. The red triangle represents the HINDEX centre and slope combination

The HINDEX cline, estimated from all 794 SNPs and representing the average genetic cline across the hybrid zone, exhibited a centre at 5,597 m, a width of 4,982 m, a slope value of 0.00015, near fixation on the left side and a value of 0.8 on the right (Figure 3 and Table S1). We observed a large variety of cline shapes for the 122 diagnostic loci (Figure 3). Most loci returned a relatively similar estimate for the centre of the cline, for example 60% ranging from 3,000 to 8,000 m. On the other hand, cline slope varied by nearly three orders of magnitude among loci, from almost 0 to 0.0027, with 66% of the loci exhibiting cline slopes higher than the HINDEX (Table S1). Cline width was strongly correlated with cline slope and varied from a few hundreds to several thousands of metres depending on the locus. Interestingly, we observed a strong relationship between centre and slope estimates, with the loci showing the sharpest clines being all centred at around 5,500–5,800 m (Figure 3).

The mean delta of cline tail values was relatively high (∆*P*_mean_ = 0.88), highlighting the fact that the 122 diagnostic loci, which are highly differentiated between the two species at large geographic scale, also show a strong turnover along the hybrid zone. A total of 56 of these loci even returned a cline with complete change in allelic frequency across the transect (∆*p* = 1), revealing that 46% of the species‐specific alleles analysed here are not exchanged between the two extremities of the hybrid zone. Furthermore, although 87% of the markers were fixed to 0 on the left side (*i.e.* “*C. macromma* side”) of the cline, only the fixed 56 reached 1 on the right tail (*i.e.* “*C. gardetta* side”). A subset of 23 loci exhibited a particularly sharp transition along the contact zone with an allelic frequency ranging from 0 to 1 in less than 3,000 m and a slope superior to 0.0005 (Figure 3). The consensus sequences of these loci were aligned against the ncbi nucleotide database using blastn, and ten of them matched Lepidoptera genes with high homology (>80%, E‐value < 10^–9^; Table 2). Among these, we detected a putative olfactory receptor, and genes involved in cell regeneration processes, in oxidative stress and in eyespot formation.

**Table 2:** Characteristic of the genes strongly differentiated between *Coenonympha macromma* and *C. gardetta* that show the sharpest clines across the hybrid zone and that match other lepidopteran genes in ncbi database

Locus ID	Organism	Gene (predicted)	Putative function	Locus accession	% identity	E‐value
CLocus_1159	*Papilio machaon*	G protein‐coupled Mth 2‐like	Olfactory receptor	XM_014503013.1	83.824	8.70E−10
CLocus_1160	*Galleria mellona*	Baculoviral IAP repeat‐containing protein 6 like (BIRC6)	Immunity	XM_026902591.1	83.333	2.66E−16
CLocus_5199	*Bicyclus anynana*	Protein AF‐10	Eyespot development	XM_024081740.1	84.783	6.26E−18
CLocus_4520	*Bicyclus anynana*	Secreted protein acidic rich in cysteine (SPARC)	Immunity	XM_024088674.1	94.118	3.69E−27
CLocus_3650	*Bicyclus anynana*	Rapamycin‐insensitive companion of mTOR (RICTOR)	Cell growth regulation	XM_024085403.1	83.333	1.79E−18
CLocus_3620	*Bicyclus anynana*	Calcium‐binding mitochondrial carrier protein SCaMC‐2	Cell protection against oxidative stress	XM_013333945.1	85.714	2.66E−16
CLocus_1941	*Vanessa tameamea*	SET and MYND domain‐containing protein 4‐like	Cell growth regulation	XM_026644751.1	82.609	3.04E−09
CLocus_1630	*Papilio polytes*	Spatacsin	Larval colour pattern	XM_013290726.1	84.783	6.26E−18
CLocus_102796	*Bicyclus anynana*	Pre‐mRNA cleavage complex 2 protein Pcf11‐like	Transcription regulation	XM_024086011.1	97.297	1.57E−25
CLocus_112938	*Bicyclus anynana*	exocyst complex component 4‐like	Exocytosis	XM_024082099.1	89.474	7.14E−11

Note

The percentage of homology, the putative function and the species for which these genes have been previously identified are shown.

### 3.4 Variation in morphological traits

**Figure 4:**
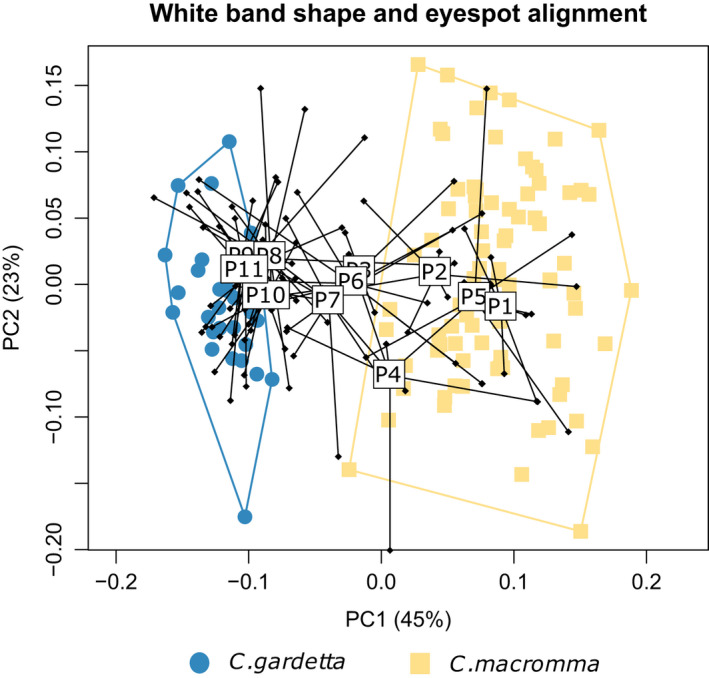
Morphological differentiation based on geometric morphometric analysis of two hindwing traits: the white band shape and the eyespot alignment. The individuals from the contact zone are shown by the black dots, and a box with the name of the population shows the barycentre of each of the 11 populations

Out of the four morphological traits measured, three strongly differed between *C. macromma* and *C. gardetta*: the relative size of the wings (hind‐ and forewing centroid size combination, the *t* test returned a *p*‐value < .001), the alignment of eyespots and the shape of the white band on the ventral side of the hindwing (MANOVA test returned a p‐value < 2.2 × 10^–16^ for the two traits) (Figure S4). When analysing together the variation of the white band shape and the eyespot alignment in a PCA, we can observe a very strong differentiation between *C. macromma* and *C. gardetta* pure populations (Figure 4), with no overlap between individuals scores along PC1 (45% of variance explained). At the opposite, the populations from the contact zone exhibit a gradual range of scores along the same PC1, ranging from values close to pure *C. macromma* individuals for P1, P2 or P5, to values close to pure *C. gardetta* individuals for P8, P9, P10 and P11. Similar to the pattern observed for the genetic composition, populations P6 and P7 show very intermediate scores on the PCA performed with these two hindwing morphological traits. By contrast, the pattern of wing venation does not seem to differ between the two species, neither for the fore‐ nor for the hindwing (Figure S4).

**Figure 5:**
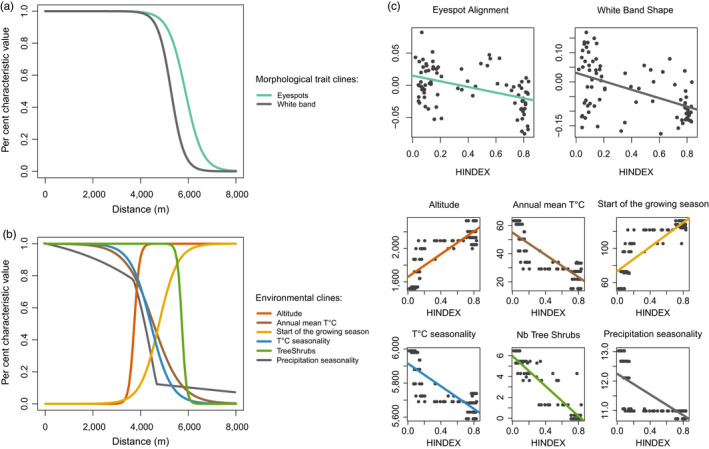
Plot of the per cent characteristic value versus. distance for two morphological traits (a) and 5 environmental variables (b) describing the sampled hybrid zone. Each line is the maximum‐likelihood fundamental cline for the best‐fit model from the seven‐model comparison. Panel (c) shows the variation of the different morphological and environmental traits along the HINDEX values with the lines showing the trends predicted by the associated linear regressions (*p*‐value and *R* ^2^ in Table 3)

Wing size returned a flat cline indicating random variations across the contact zone (Figure S6). In contrast, eyespot alignment, summarized by the individual scores along the first axis of a PCA performed on the eyespot centre landmarks (66% of variance explained), and white band shape, summarized by the individual scores along the first axis of a PCA performed on the white band landmarks (47% of variance explained), showed cline variations that coincide with the estimated centre of the genetic cline (Table 3 and Figure 5). Cline centre was located around 5,940 m for eyespots and around 5,260 m for the white band. The ML estimate for cline width was larger for eyespots (8 km) than for the white band (1.1 km). The linear regression between individual HINDEX estimates and morphological traits confirmed the association between genetic and morphological variation along the hybrid zone, pointing out to a stronger association between individual genetic composition and variation in the white band (*r* ^2^ = .46) than for eyespots (*r* ^2^ = .25).

**Table 3:** Names and maximum‐likelihood parameter estimates of the centre, width and slope of the best‐fit cline model for HINDEX, morphological traits and environmental variables

	Cline model	Centre	Width	Slope	*p*‐value	*R* ^2^
Genetic						
Hindex	Free bounds and none fitted tails	5,597	4,982	0.00015	–	–
Morphology						
Eyespots	Free bounds and none fitted tails	5,938	8,465	0.00003	3.6e−05	0.25
White band	Free bounds and none fitted tails	5,262	1,174	0.00033	1.3e−06	0.46
Environment						
Altitude	Free bounds and none fitted tails	4,566	690	0.00099	<2e−16	0.71
Annual mean T¡C	Free bounds and both tails estimated separately	4,520	2,357	0.00036	<2e−16	0.69
Start of the growing season	Free bounds and none fitted tails	4,751	1,604	0.00051	<2e−16	0.74
Precipitation seasonality	Free bounds and both tails estimated separately	4,591	1,612	0.00125	9.8e−16	0.52
T¡C seasonality	Free bounds and both tails estimated separately	4,366	1,918	0.00043	<2e−16	0.67
Nb of Tree & Shrubs	Free bounds and none fitted tails	5,482	358	0.00222	<2e−16	0.9

Note

The last two columns give the *p. value* and *r‐squared* (*R* ^2^) of a linear regression between the HINDEX and the morphological traits (sample size: *n* = 81 individuals), and between the HINDEX and the environmental variables (*n* = 93 individuals) across the hybrid zone.

### 3.5 Pattern of ecological transition along the hybrid zone

Most of the environmental variables tested showed a significant difference between the locations of the pure populations of both *C. macromma* and *C. gardetta* (Figure S7). Only the maximum NDVI, a proxy for vegetation productivity, was not significantly different between species‐specific locations, and elevation was only marginally significant (*p*‐value .04). Furthermore, all the environmental variables that distinguish the two parental species showed a strong cline across the contact zone, with ML estimates of cline centre close to those obtained for genetic markers and morphological traits (4,300 m for annual mean temperature, 5,480 m for the proportion of trees and shrubs) (Figure 5 and Figure S8). The width of the clines varied from only 350 metres for the proportion of trees and shrubs to more than 2 km for the annual mean temperature (Table 3). The proportion of trees and shrubs, a variable characteristic of the habitat, showed the sharpest transition along the hybrid zone and returned a cline centre estimate (5,480 m) very close to the one obtained for the HINDEX (5,597 m). In the same way, the strongest statistical association was found between individual genetic composition (HINDEX) and the proportion of high vegetation structure along the hybrid zone (*r* ^2^ = .9; Table 3).

## 4 DISCUSSION

### 4.1 An active, narrow and long‐lasting hybrid zone

Our study confirmed the presence of an active hybrid zone between *C. macromma* and *C. gardetta* in the south of the French Alps (near Col de Vars, Hautes‐Alpes, France). We characterized in this area a set of 11 populations showing a gradient of admixture between pure *C. macromma* individuals and nearly pure *C. gardetta* individuals (Figures 1 and 2). Reproductive isolation is clearly not complete between these species, in agreement with a previous study, showing that the two taxa had undergone an uninterrupted history of contact and gene flow since their initial divergence 12,000–20,000 years ago (Capblancq et al., 2019). Yet, the two species keep their genetic integrities at large scale and the admixture found in this study remains restricted to the centre of a narrow hybrid zone (Figure 1; Capblancq et al., 2019).

The SNP cline analysis revealed a rapid genetic transition along a hybrid zone less than 9 km wide (Figure 3). Such a sharp genetic transition could reflect a recent secondary contact between the two species (i.e. ephemeral‐zone hypothesis; Moore, 1977), but the low level of interspecific heterozygosity in hybrids does not support this hypothesis and rather suggests an advanced‐generation hybrid zone (Figure 2). Simulation‐based and empirical studies have shown that a hybrid zone with various degrees of individual admixture combined with low interspecific heterozygosity results from a long‐lasting contact and widespread recombination between species (De La Torre et al., 2015; Fitzpatrick, 2012; Lindtke et al., 2012). Although RAD‐like experiments can underestimate heterozygosity (Cariou, Duret, & Charlat, 2016) and could have biased our results to a certain extent, such experimental bias cannot explain the very low level of interspecific heterozygosity found in the contact zone (Figure 2) or the small difference between parental and contact zone populations’ heterozygosity values (Table 1). In a previous study, we found high levels of interspecific heterozygosity for individuals of the same complex of species, genotyped with the same ddRADseq experiment and analysed the same way (Capblancq et al., 2019), showing that the low interspecific heterozygosity found in the hybrid zone cannot be only due to experimental and/or bioinformatics treatments.

### 4.2 Genetic architecture of introgression between *C. macromma* and *C. gardetta*

Hybrid zones are expected to show variation in introgression rates along the genome, with reduced introgression for genomic regions involved in disruptive selection, whereas neutral regions can be exchanged freely between species (Barton & Hewitt, 1985; Nosil, Funk, & Ortiz‐Barrientos, 2009; Rieseberg et al., 1999). The regions displaying restricted circulation across the hybrid zone are therefore expected to be under disruptive selection and likely correspond to genomic regions responsible for genetic incompatibilities, prezygotic isolation or involved in local adaptation to differences in the environment (Gompert, Lucas, Fordyce, Forister, & Nice, 2010; Walsh, Gregory Shriver, Olsen, & Kovach, 2016). Thus, the investigation of the genetic architecture of hybrid zones provides a way to estimate the nature of the selective agents driving the divergence between species and shed light on their patterns and strengths along the genome and across the landscape (De La Torre et al., 2015; Ravinet et al., 2017; Stankowski et al., 2017).

In the hybrid zone studied here, patterns of variation in the estimates of cline centre and slope revealed some heterogeneity in cline shapes. Cline slope estimates are highly variable among the 122 diagnostic loci, although most loci show slope estimates below the global genetic cline value (HINDEX), whereas a few show extremely strong values (Figure 3b). In the same way, the centre of the cline estimates can vary greatly along the hybrid zone, but the steepest clines are all centred around 5,600 m (Table S2). This kind of heterogeneous genomic divergence has already been described for many other species pairs (Fitzpatrick et al., 2009; Payseur, 2010; Payseur, Krenz, & Nachman, 2004; Rieseberg et al., 1999), especially when the process of speciation began recently (Nosil et al., 2009). Given that *C. macromma* and *C. gardetta* have likely been diverging for only a few thousands of years (12,000‒20,000 years; Capblancq et al., 2015), it is not surprising to find such pattern in this hybrid zone, even if a quantitative association between divergence time and strength of isolation is not trivial when we deal with hybrid species (Mallet, 2007). Indeed, due to the reshuffling of parental traits and genomes, even recent hybrid species can be strongly isolated from parents (Lukhtanov, Shapoval, Anokhin, Saifitdinova, & Kuznetsova, 2015; Nice et al., 2013; Schwander, Suni, Cahan, & Keller, 2008; Schwarz, Shoemaker, Botteri, & McPheron, 2007). In agreement with this assertion, there is probably a nearly complete reproductive isolation between *C. macromma* and its other parental species, *C. arcania,* since only one natural hybrid has been found in a previously studied contact zone between these two species (Capblancq et al., 2019), although, according to the hybrid speciation scenario, they would have diverged for the same number of generations (12,000–20,000 years/generations).

Along the hybrid zone, some markers do not reach a *P*_max_ = 1 in the right tail of their clines (*i.e.* “*C. gardetta* side”), even when scored several kilometres away from the centre (P11 in Figure 3a). At the opposite, almost all the makers return a *P*_min_ of 0 in the left side of the cline (*i.e.* “*C. macromma* side”). It suggests that a part of *C. macromma* genetic material can introgress more easily into the *C. gardetta* genetic background than the reverse. Such asymmetry in the introgression pattern has already been associated with differences in population abundance (Lepais et al., 2009), with hybrid zone movement due to climate change (Taylor et al., 2014), or with scenarios in which the introgressive population is expanding while the introgressed population is declining (Johnson, White, Phillips, & Zamudio, 2015). A deeper investigation would be necessary to understand the causes of this pattern in the studied hybrid zone, although abundance does not seem to strongly differ between species in the sampled localities (field observations).

Almost half of the 122 diagnostic loci between *C. macromma* and *C. gardetta* seem to experience a restricted allelic transfer across the hybrid zone: the mean allelic frequency change along the hybrid zone is 0.88, with 46% of the loci even showing a complete frequency change (Figure 3 and Table S1). These 122 diagnostic loci also show cline width estimates not exceeding the spatial extent of the hybrid zone. Altogether, these results suggest that a portion of the genome is nearly impermeable to gene flow between the two species and that recombination of these genomic regions is restricted to the core of the hybrid zone. The degree of reproductive isolation between the species, if not complete, would thus be at least relatively strong, confirming the genetic integrity of species at a large geographic scale (Capblancq et al., 2015, 2019).

### 4.3 Drivers of species isolation

The abrupt geographic transition observed between the genetic makeup of the two species and the unimodal distribution of admixed genotypes in the core of the hybrid zone could be consistent with two types of described hybrid zone models: a tension zone model (Buggs, 2007; Jiggins & Mallet, 2000; Wielstra et al., 2017) or a bounded hybrid superiority model (Abbott, 2017; Moore, 1977). In the tension zone theory, hybrid zones are maintained by a balance between species dispersal and selection against hybrids due to genetic incompatibilities between parental genomes (Barton, 2001; Barton & Hewitt, 1985). The sharpness of the genetic clines seen here would thus reflect strong selective constraints and/or low dispersal capacity of the species (Arias et al., 2012). Again, the low interspecific heterozygosity observed in this hybrid zone suggests a long‐term and “stabilized” hybrid zone and, together with its narrow width, makes it unlikely that a poor dispersal capacity alone could prevent the complete mixing of the genomes in the area (Barton & Hewitt, 1989). Besides, it is unlikely that hybrids show a strongly decreased fitness given their abundance in the core of the hybrid zone (field observation). On the other hand, the bounded hybrid superiority model states that strong disruptive selection mediated by behavioural or environmental factors could participate to shape hybrid zone dynamics (Abbott, 2017; Moore, 1977). In this case, hybrids would be more fit than parents in the core of the hybrid zone but restricted to this particular ecotone (Grant, Grant, Keller, Markert, & Petren, 2003; Mallet, 2005). Considering the number of diagnostic loci showing a very steep cline along the hybrid zone (56/122), it would suggest that various regions of the genome are involved in environmental disruptive selection. However, genomic regions involved in ecological adaptation are usually restrained to few parts of the genome (Nosil et al., 2009), although polygeny and epistasis can also be involved in ecological divergence (Albert et al., 2008; Arnegard et al., 2014; Des Roches, Sollmann, Calhoun, Rothstein, & Rosenblum, 2017).

Here, our results suggest a more complex interplay of different types of barriers to reproduction, partially associated with the particularity of the hybrid origin of *C. macromma*. We found a great concordance between genetic, morphological and environmental variations along the hybrid zone (Figure 3 and Figure 5), as often observed in natural hybrid zones involving recently diverged animal species (Arias et al., 2012; Rosser, Dasmahapatra, & Mallet, 2014; Taylor et al., 2014). Both intrinsic barriers (e.g. assortative mating, habitat choice) and extrinsic barriers (e.g. local adaptation) could be involved in the reproductive isolation between *C. macromma* and *C. gardetta*. Among the loci least permeable to introgression in the hybrid zone, some are homologous to genes involved in olfactory receptors, eyespot development, immunity and cell protection against oxidative stress in other butterfly species. The more divergent genomic regions between *C. macromma* and *C. gardetta* may thus include genes putatively involved in assortative mating through wing pattern or pheromone recognition, or adaptations to stressful environmental conditions.

#### 4.3.1 Assortative mating

Highly visible wing patterns such as the white band and the eyespots of the ventral side of the hind‐wings had steeper clines and were far more concordant with the genetic clines than wing size or venation patterns (Figure 5). This suggests the action of divergent selection on wing patterns that are visually different between the species, probably as a consequence of visual mate choice favouring assortative mating. In agreement with this hypothesis, one of the genomic regions showing the lowest introgression rate between the two species corresponded to a gene involved in eyespot development in *Bicyclus anynana* (Özsu, Chan, Chen, Gupta, & Monteiro, 2017). Further support for assortative mating as a source of isolation in this species pair comes from the observation that another genomic region with very low introgression rate corresponded to a putative olfactory receptor in *Papilio machaon*, suggesting that volatile organic compounds might be involved in chemical signalling and mate choice in *Coenonympha*. The involvement of both visual and olfactory cues in partner choice and species isolation has been demonstrated in many nymphalid butterflies (Costanzo & Monteiro, 2007; Mallet, Owen McMillan, & Jiggins, 2006; Mavárez et al., 2006; Mérot, Frérot, Leppik, & Joron, 2015; Pinzari et al., 2018). Although indirect, our results suggest that both visual and chemical signals might be involved in *Coenonympha* species reproductive isolation.

#### 4.3.2 Ecological divergence

Our results show that *C. gardetta* individuals are mostly found in open alpine grasslands, while *C. macromma* tend to be found in mixed habitats in which trees and large shrubs cover a significant fraction of the area. Indeed, the amount of high vegetation is the environmental factor most strongly associated with clinal genetic variation in this hybrid zone (Table 3). Alpine species have not only to handle with harsh climatic conditions including snow, low temperatures and storms, but they also have to face more wind, solar radiation, rapid desiccation and ample daily temperature variation in the absence of protective vegetation structures (Huey, 1991; Sinclair, Roger Worland, & Wharton, 2003; Turlure, Choutt, Baguette, Hans, & Dyck., 2010). This could explain some of the ecological differences between *C. macromma* and *C. gardetta,* which are both adapted to high‐elevation conditions but do not seem to use the same local habitats. Here again, several genetic markers found to be highly species‐specific across the hybrid zone could be involved in this environmental‐divergent adaptation, including a gene involved in cell oxidative stress, which is supposedly higher at full solar radiation than under vegetation cover (Meng, Zhang, Zhu, Wang, & Lei, 2009). Several genes involved in cell growth regulation/transcription might also be involved in differential physiological responses to stress‐full conditions in open light (UV damages) as opposed to the possibility for *C. macromma* to behaviourally avoid these damages and overheating by hiding within high vegetation. In the same vein, it has been shown for another butterfly of the same genus, *C. pamphilus,* that vegetation structure, as a mating place, is key for reproductive success (Wickman, 1992). If a similar process drives habitat choice for *C. macromma*, the lack of high vegetation in high‐elevation open meadows could be a strong barrier to hybridization with *C. gardetta*.

#### 4.3.3 Genetic incompatibilities

None of our results can confirm or reject the presence of genetic incompatibilities between *C. macromma* and *C. gardetta*. However, several genes showing low or no introgression between the two genomes involve basic general cellular processes such as cell growth or transcription regulation: these processes typically involve many interacting genes. Barrier effects in these genes can evolve through the spread of incompatible alleles via drift in the two diverging populations. Intrinsic incompatibilities could also evolve as a by‐product of divergent ecological selection (Kulmuni & Westram, 2017). Indeed, genes can have multiple functions (pleiotropy) and/or function in complex networks, and their evolution is not independent of other genes: changes evolving in response to ecological selection can generate intrinsic barriers as a by‐product.

Altogether, our results suggest that the abrupt geographic transition observed between the two genetic pools is shaped by the action of various ongoing selective pressure(s) that maintain the species integrity despite incomplete isolation and many generations of genetic exchange. We go a step further in dissecting the barrier effects, and found that both intrinsic (assortative mating through visual and olfactory cues) and extrinsic (vegetation structure) barriers are probably involved in reproductive isolation between *C. macromma* and *C. gardetta*, with various genomic regions involved, and several putative candidate genes are identified. The sharpness of the clines fitted for those genomic regions suggests ongoing selection. However, neutral markers can also exhibit very sharp clines under the effect of strong genetic drift increasing the probability of fixation of the major allele on both sides of the hybrid zone (Polechová & Barton, 2011). We cannot completely rule out this possibility here even if the strong concordance of the identified cline centres in the middle of the hybrid zone makes this pattern unlikely to be due to drift alone (Figure 3). Further work would thus be needed to validate the involvement of visual and olfactory cues in mate choice in these species, and to evaluate the many possible impacts of habitat structure not only on mate choice, but also on the diversity of predators/pathogens and predator‐escape strategies, and on the thermo‐regulation strategies.

### 4.4 What future for the two species in the Alps?

This study provides evidence for coincidence of morphological, ecological and genetic clines in a narrow hybrid zone where the overall barrier to gene flow is strong, but not complete. The incompleteness of the reproductive isolation observed in these two alpine taxa highlights the tenuous balance between dispersal and selection commonly observed in hybrid zones (Abbott, 2017; Barton & Hewitt, 1985, 1989; Buggs, 2007; Moore, 1977). The Alps are currently experiencing dramatic environmental changes, due to both the evolution of human activities and global warming (Lavergne, Mouquet, Thuiller, & Ronce, 2010). If, as we suggest here, the differentiation between *C. gardetta* and *C. macromma* is partially maintained by divergent environmental requirements, we might expect a disruption of the hybrid zone equilibrium in the next decades. The rise of temperature together with the increase in shrub/tree coverage at high elevations could favour *C. macromma* over its parental species *C. gardetta*, pushing the contact zone towards northernmost latitudes, as already reported for many other hybrid zones (Buggs, 2007; Ryan et al., 2018; Taylor et al., 2014; Thurman, Szejner‐Sigal, & Owen McMillan, 2019; Wielstra et al., 2017). More dramatically, an unconstrained introgression of *C. macromma* genetic material into *C. gardetta* genetic pool would threaten the genetic integrity of the species in the future, at least locally (Hohenlohe, Amish, Catchen, Allendorf, & Luikart, 2011; Vonlanthen et al., 2012). Monitoring the potential change that will happen in this hybrid zone would thus be of critical importance to envision the future of this particular couple of species and could, more generally, act as a window on biodiversity threat in the Alps (Taylor, Larson, & Harrison, 2015; Wielstra, 2019). Our ability to predict such changes will be key to future conservation and management efforts.

## CONFLICT OF INTEREST

None declared.

## AUTHOR CONTRIBUTIONS

J. Mavarez, L. Després and T. Capblancq designed the study and collected the samples. T. C. performed ddRAD experiments and bioinformatics treatment of the sequences, as well as the genetic, morphologic and environmental analyses. L.D. performed the ncbi blast analysis. T. C., J. M. and L. D. wrote the manuscript.

## Data Availability

Genetic data in the form of a table of genotypes for each individual and each retained site are available at Dryad Digital Repository (https://doi.org/10.5061/dryad.4mj8gg0). Morphologic data in the form of the coordinates of the 40 morphometric landmarks for each individual are available at Dryad Digital Repository (https://doi.org/10.5061/dryad.4mj8gg0). Environmental data in the form of climatic rasters and a table of habitat variables are available at Dryad Digital Repository (https://doi.org/10.5061/dryad.4mj8gg0). The ProcessMyRAD pipeline is freely available on Github at https://github.com/cumtr/PmR. Scripts used to perform the different analyses are freely available on Github at https://github.com/Capblancq/Speciation-Coenonympha-butterflies. Digitalized pictures of the butterfly wings are freely available upon request from T. Capblancq.
