# Supplementary figures and images for: Genetic, morphological and ecological variation across a sharp hybrid zone between two alpine butterfly species
Source: Evol Appl. 2020 Feb 7;13(6):1435–50. doi: 10.1111/eva.12925 (PMC7359832; doi:10.1111/eva.12925)

## Proportion of polymorphic stacks

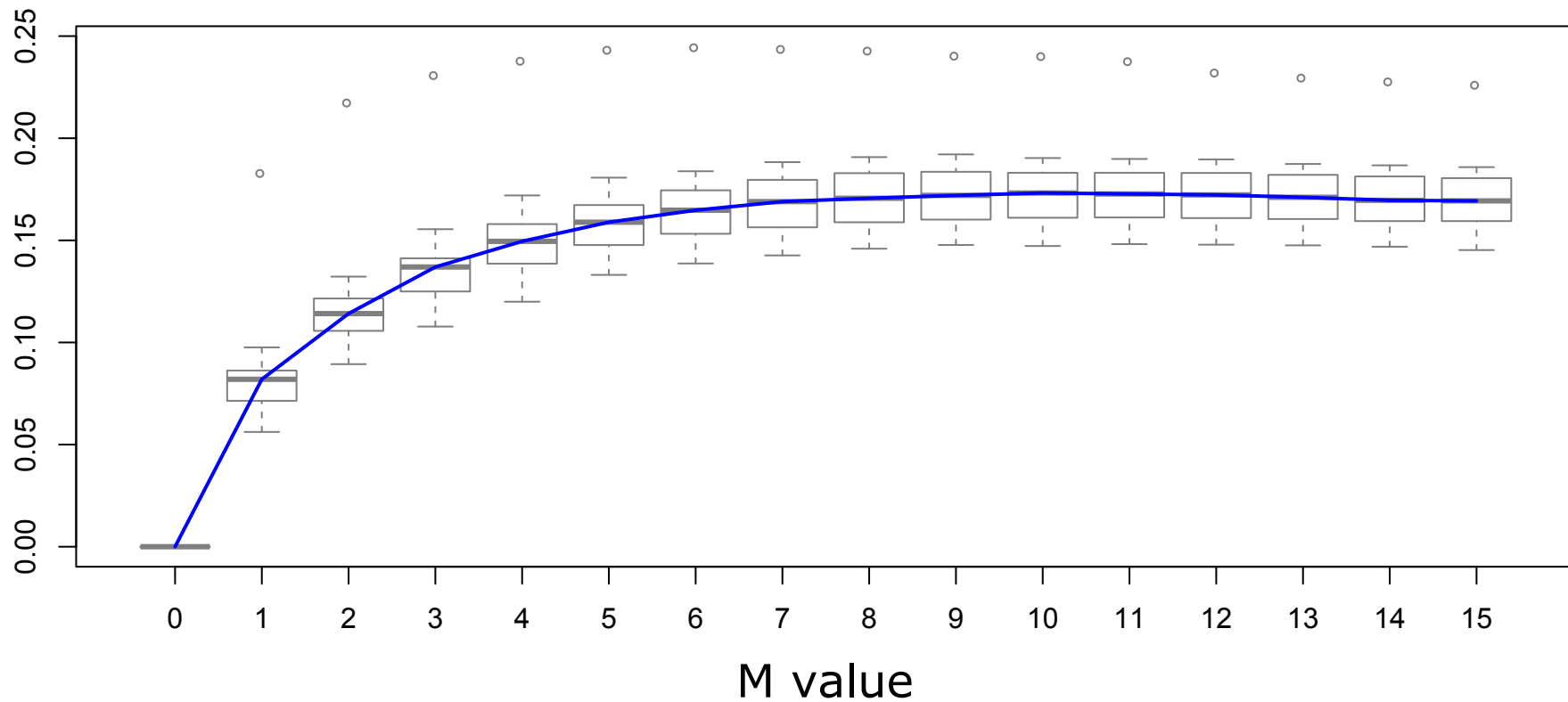

Supplement: Supplementary file 1 [file EVA-13-1435-s001.pdf]

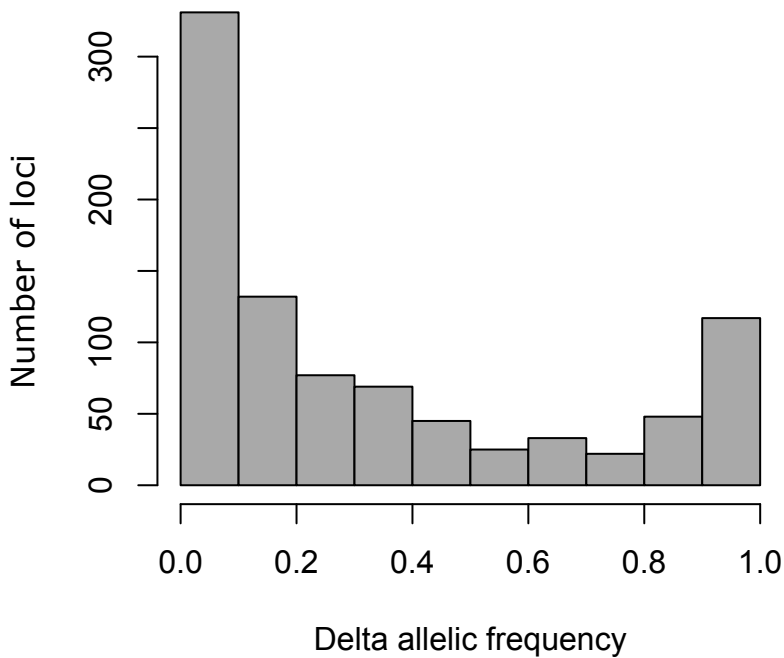

Supplement: Supplementary file 2 [file EVA-13-1435-s002.pdf]

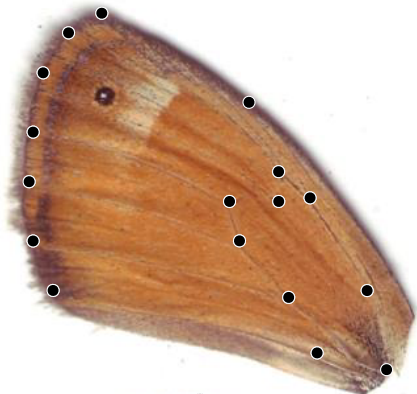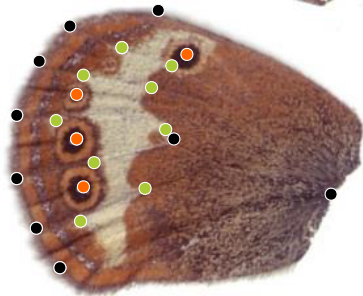

- White-band shape
- Eyespots alignment
- Venation

Supplement: Supplementary file 3 [file EVA-13-1435-s003.pdf]

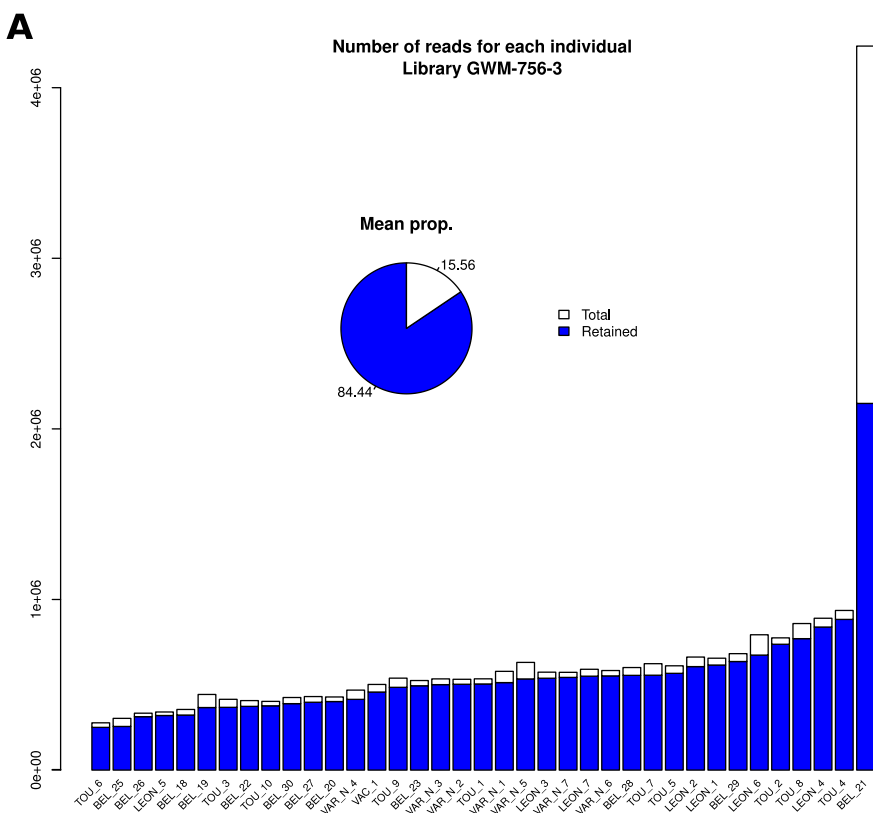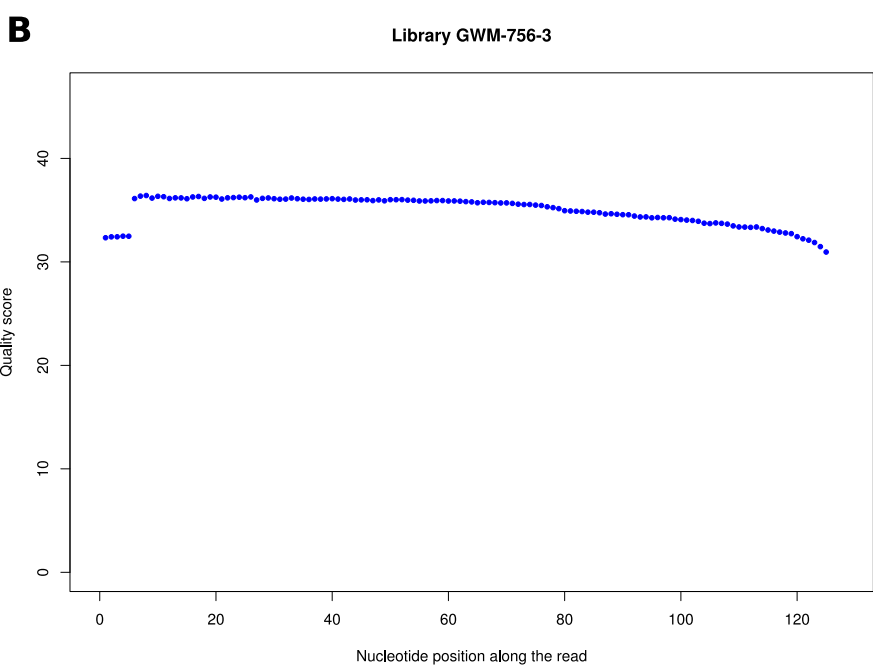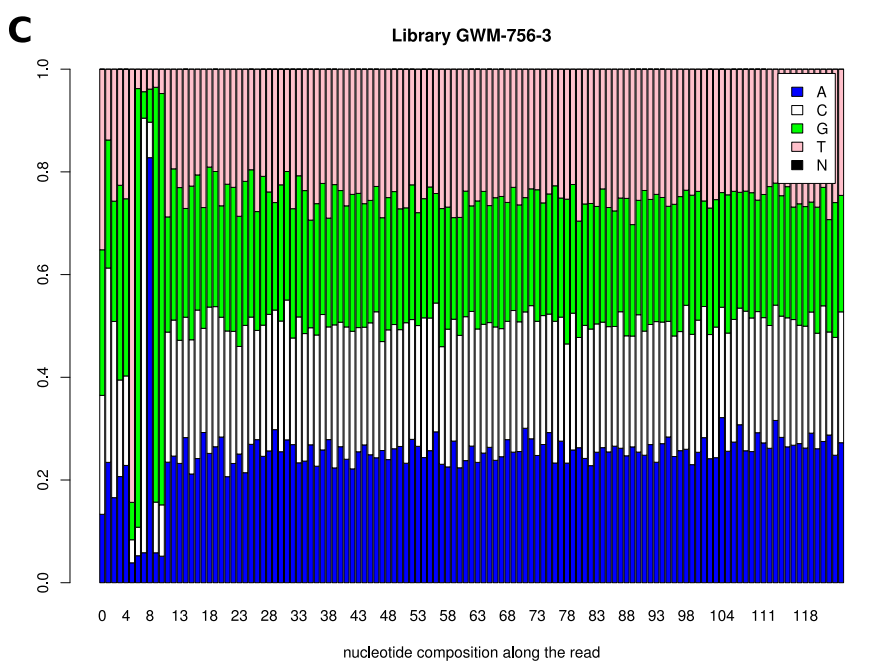

Supplement: Supplementary file 5 [file EVA-13-1435-s005.pdf]

**Eyespots**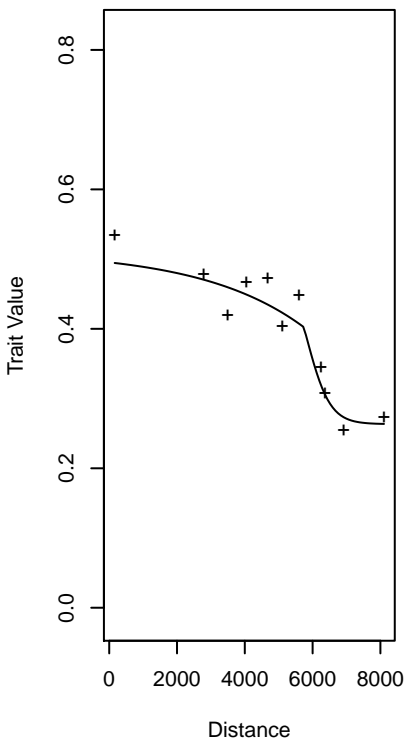**Whiteband**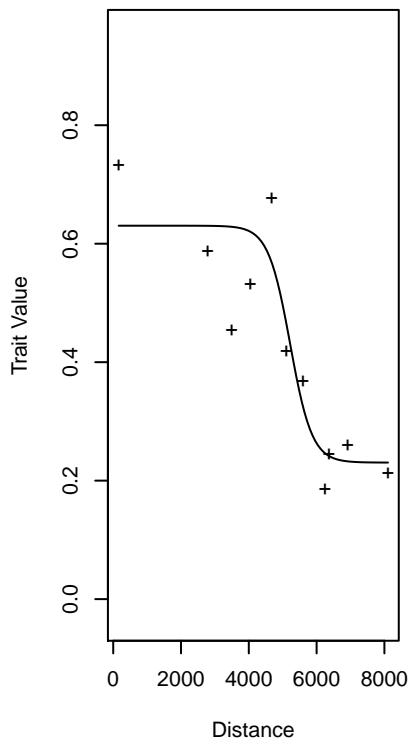**WingSize**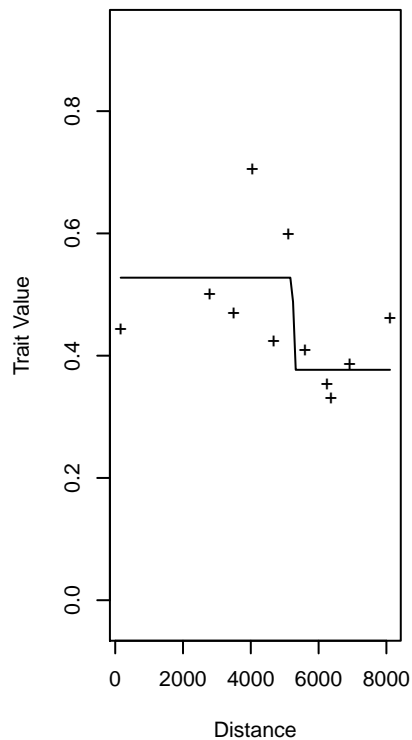

Supplement: Supplementary file 6 [file EVA-13-1435-s006.pdf]
