# Supplementary material for: Genetic, morphological and ecological variation across a sharp hybrid zone between two alpine butterfly species
Source: Evol Appl. 2020 Feb 7;13(6):1435–50. doi: 10.1111/eva.12925 (PMC7359832; doi:10.1111/eva.12925)
Supplement: Supplementary file 4 [file EVA-13-1435-s004.pdf]

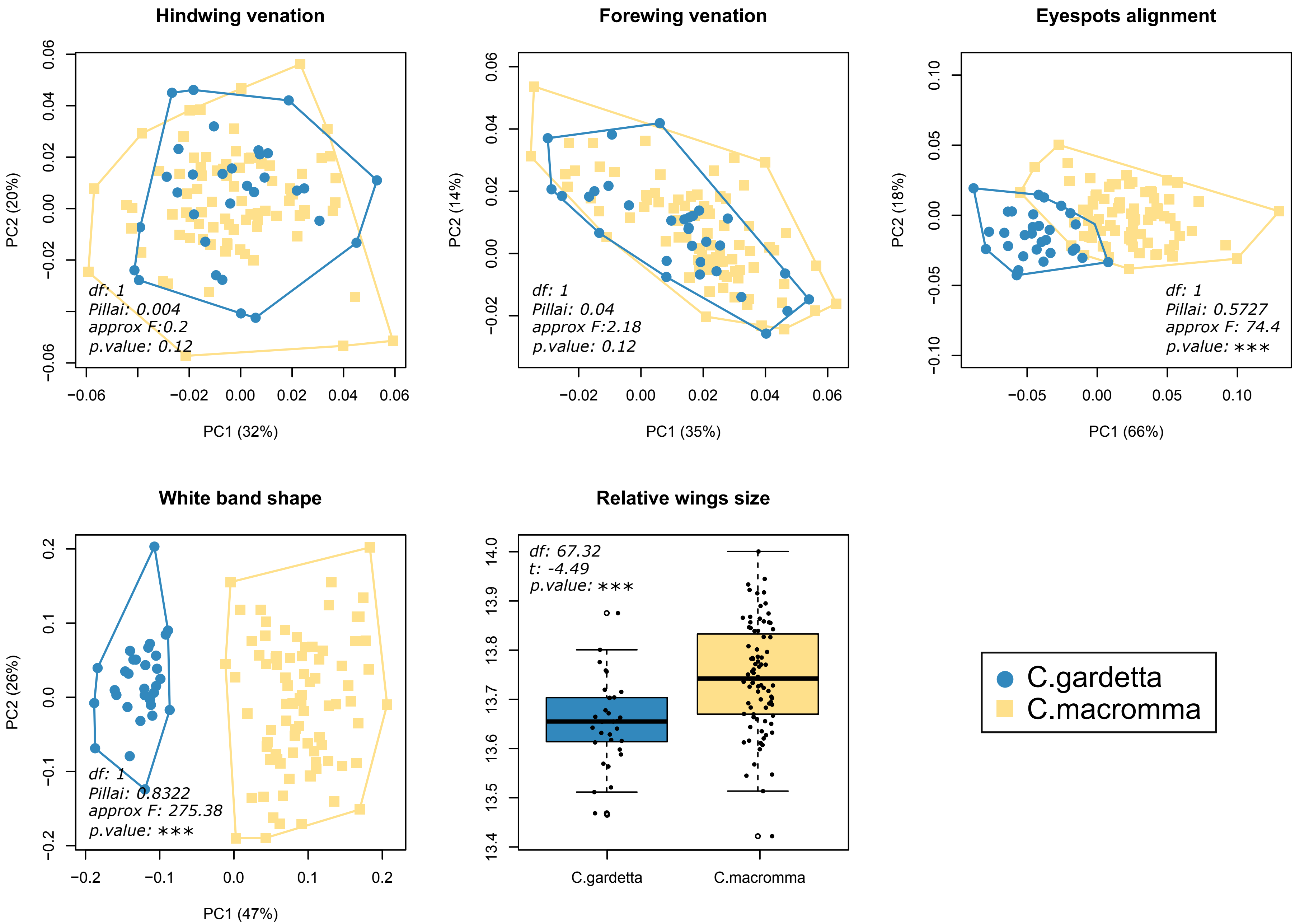

Hindwing venation
PC1 (32%)
PC2 (20%)
df: 1
Pillai: 0.004
approx F:0.2
p.value: 0.12
Forewing venation
PC1 (35%)
PC2 (14%)
df: 1
Pillai: 0.04
approx F:2.18
p.value: 0.12
Eyespots alignment
PC1 (66%)
PC2 (18%)
df: 1
Pillai: 0.5727
approx F: 74.4
p.value: ***
White band shape
PC1 (47%)
PC2 (26%)
df: 1
Pillai: 0.8322
approx F: 275.38
p.value: ***
Relative wings size
df: 67.32
t: -4.49
p.value: ***
C.gardetta
C.macromma
