# Supplementary material for: Genetic, morphological and ecological variation across a sharp hybrid zone between two alpine butterfly species
Source: Evol Appl. 2020 Feb 7;13(6):1435–50. doi: 10.1111/eva.12925 (PMC7359832; doi:10.1111/eva.12925)
Supplement: Supplementary file 7 [file EVA-13-1435-s007.pdf]

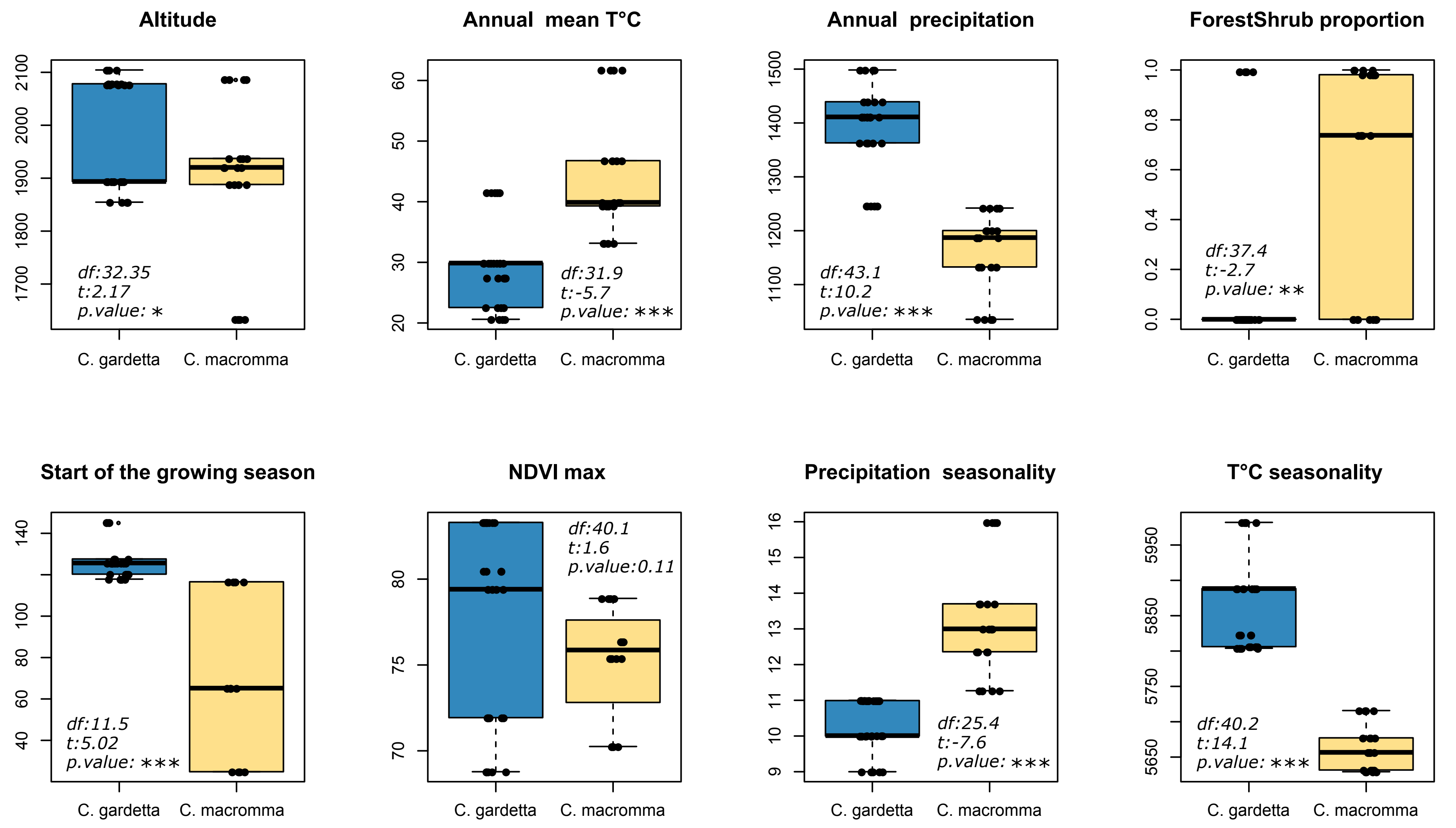

Altitude
df:32.35
t:2.17
p.value: *
Annual mean T°C
df:31.9
t:-5.7
p.value: ***
Annual precipitation
df:43.1
t:10.2
p.value: ***
ForestShrub proportion
df:37.4
t:-2.7
p.value: **
Start of the growing season
df:11.5
t:5.02
p.value: ***
NDVI max
df:40.1
t:1.6
p.value:0.11
Precipitation seasonality
df:25.4
t:-7.6
p.value: ***
T°C seasonality
df:40.2
t:14.1
p.value: ***
C. gardetta
C. macromma
