# Supplementary material for: Genetic, morphological and ecological variation across a sharp hybrid zone between two alpine butterfly species
Source: Evol Appl. 2020 Feb 7;13(6):1435–50. doi: 10.1111/eva.12925 (PMC7359832; doi:10.1111/eva.12925)
Supplement: Supplementary file 8 [file EVA-13-1435-s008.pdf]

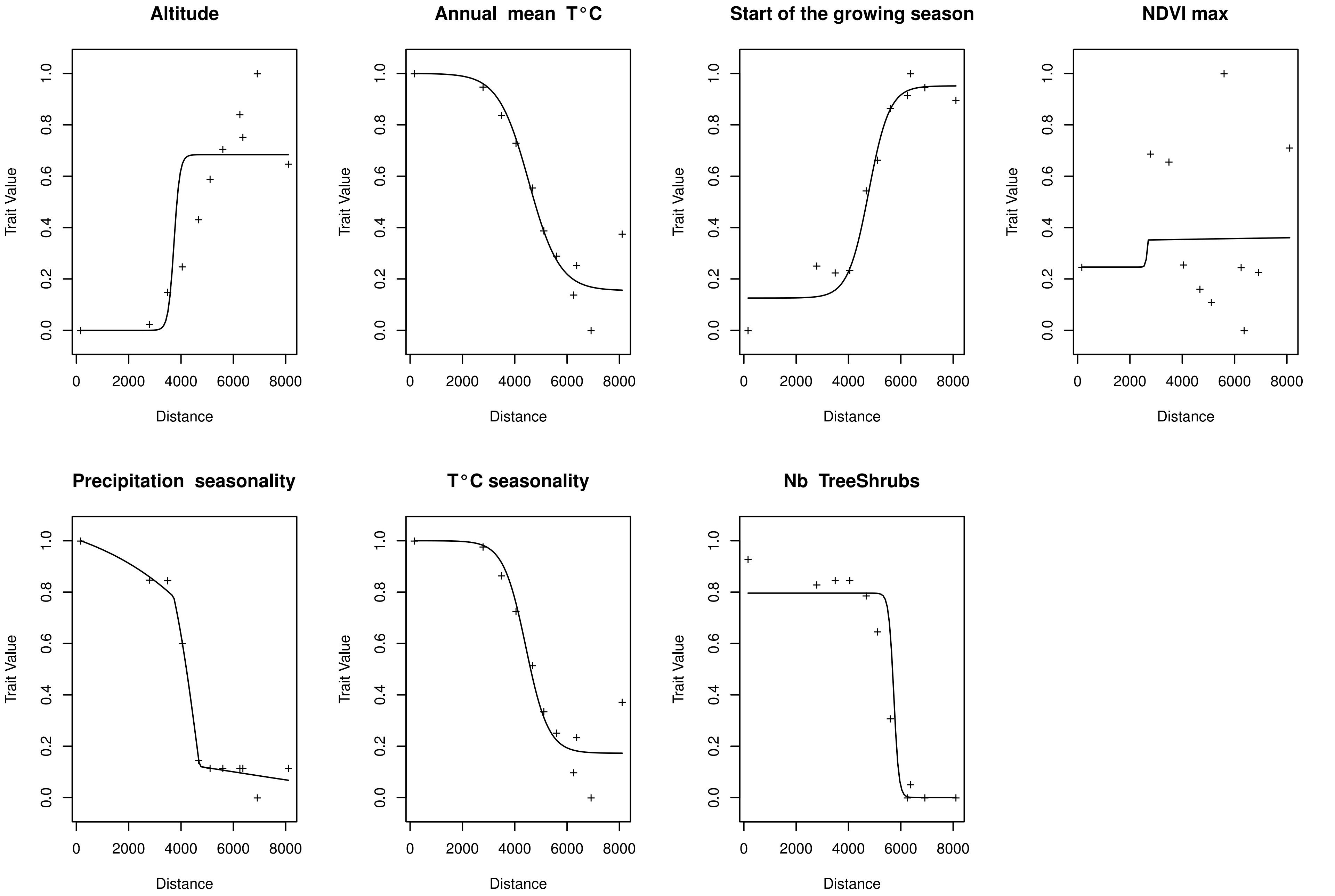

Altitude
Annual mean T°C
Start of the growing season
NDVI max
Precipitation seasonality
T°C seasonality
Nb TreeShrubs
Trait Value
Distance
0.0
0.2
0.4
0.6
0.8
1.0
0
2000
4000
6000
8000
